# Tuberculosis in Kazakhstan: analysis of risk determinants in national surveillance data

**DOI:** 10.1186/1471-2334-12-262

**Published:** 2012-10-18

**Authors:** Assel Terlikbayeva, Sabrina Hermosilla, Sandro Galea, Neil Schluger, Saltanat Yegeubayeva, Tleukhan Abildayev, Talgat Muminov, Farida Akiyanova, Laura Bartkowiak, Zhaksybay Zhumadilov, Almaz Sharman, Nabila El-Bassel

**Affiliations:** 1 Columbia University Global Health Research Center of Central Asia, 102 Luganskogo str, Almaty, 050059, Kazakhstan; 2 National Center for Tuberculosis in Kazakhstan, 5 Bekhodjin str, Almaty, 050059, Kazakhstan; 3 Kazakhstan National Association of TB Specialists, 5 Bekhodjin str, Almaty, 050059, Kazakhstan; 4 Kazakhstan National Institute of Geography, 99 Pushkin str, Almaty, 050010, Kazakhstan; 5 Nazarbayev University; address, 5 Kabanbay Batyr str, Astana, 010000, Kazakhstan; 6 6) Columbia University in the City of New York, 1255 Amsterdam Avenue, New York, NY, 10027, USA

**Keywords:** Tuberculosis, Case notification rate (CNR), Prevalence, Risk factors, MDR-TB, Kazakhstan, Spatial distribution, Surveillance, National tuberculosis program (NTP), Oblast

## Abstract

**Background:**

Development of tuberculosis (TB) is determined by various risk factors and the interactions of temporal and spatial distributions. The aim of this study was to identify the most salient risk factors for TB disease as well as multidrug resistant TB (MDR-TB) at the oblast (provincial) level in Kazakhstan.

**Methods:**

Correlational and descriptive analyses were conducted at the oblast and national level using data provided by the country’s National Institute of Geography (NIG) and the National Tuberculosis Program (NTP). Reported incident case notification rates (CNRs) and prevalence vary by oblast, thus the study investigated which determinants contributed to this regional variation and compared burdens among oblasts.

**Results:**

The results showed that while tuberculosis CNRs decreased over the study period, MDR-TB conversely increased. Two oblasts -Atyrauskaya and Mangystauskaya - presented especially significant anomalies with large decreases in TB incident CNRs coupled with comparatively large increases in MDR-TB incident CNRs.

**Conclusion:**

Understanding the distribution of TB and MDR-TB cases and associated risk factors, especially the “unknown risk factor” categorization points to the need for future research.

## Background

Despite World Health Organization’s (WHO) recent report describing a decline in tuberculosis (TB) morbidity and mortality, the disease remains an acute threat to global public health [1]. The increase in tuberculosis case notification rates (CNRs) across much of the former Soviet Union (FSU) since its dissolution has been well-documented [2,3]. Following the break-up of the Soviet Union, Kazakhstan experienced a dramatic increase in tuberculosis infections. Incidence rates reported to the WHO in 1995 (135/100,000 population), 2000 (196/100,000), and 2004 (223/100,000) chronicled this increase, which spurred the country’s public health sector to introduce new approaches to tuberculosis control [4]. Starting in 1998, Kazakhstan’s Ministry of Health (MoH) began implementing Directly Observed Therapy Short Course (DOTS) through the then newly created National Tuberculosis Program (NTP), which continues to oversee TB care and treatment in the country today.

In several FSU republics including Kazakhstan, multidrug resistant forms of the disease increasingly threaten TB control efforts [5,6]. The NTP provides free TB treatment for its residents via a centralized network of TB dispensaries, hospitals, and polyclinics. While polyclinics may perform tentative TB diagnosis and later serve as local sites for continued treatment, they initially refer all suspected cases to NTP facilities [7]. The NTP system currently consists of 315 microscopy laboratories and funding was recently allocated to the network to improve diagnostic capacities. In the first quarter of 2012, the NTP reported that Kazakhstan was the only Central Asian republic where all of the oblasts had the capacity to perform drug resistance testing, citing a testing rate of 98.2% of new TB cases and 98.9% of retreatment cases covered by DOTS [8]. However, TB drug resistance testing of all cases has not been fully achieved within Kazakhstan’s prison system. Hein genotyping analysis is currently being piloted in 10 out of 14 oblasts. Despite these advances in TB control efforts, in practice, TB diagnosis is often based on x-ray rather than bacteriological analysis.

TB incidence peaked in Kazakhstan in 2003 and 2004, since then the NTP has attained some success in restraining further increases; however, MDR-TB presents a growing challenge to controlling TB. The WHO reports that 14% (range 11-18%) of all newly diagnosed TB cases in Kazakhstan are multidrug resistant. Among retreatment cases, 45% test positive for MDR-TB [4]. Since such statistics were first recorded in 1998, the country’s success rates for treatment of new TB cases have consistently fallen below the WHO-recommended ≥85% cure rate. In fact, success rates declined to 62% in 2009 after achieving an earlier high of 79% [9,10]. The problem is compounded by high MDR-TB rates in prisons, where CNRs may be five times higher than in the general population [11].

Interest in the social, economic, and environmental determinants of TB has grown as CNRs have risen during the past two decades. The contextual milieu in which individuals live and work is critical to understanding and stemming the disease [12]. A literature review identified several global risk factors for TB. Individual risks include age, sex, smoking, alcoholism, diabetes, HIV status, marital status, ethnicity, homelessness, incarceration, drug use, and migrant status [13-18]. Socioeconomic and environmental risk factors take into account deprivation, financial insecurity, and housing conditions. [13,14,19] While these risk factors may play a role in increasing Kazakhstan’s overall TB burden and intensifying MDR-TB, they have not been adequately examined.

This study presents the first comprehensive description of the spatial and temporal burden of TB disease constructed from data obtained from the Kazakhstani national TB surveillance system. Study findings identify salient risk factors for TB disease as well as MDR-TB at the oblast (provincial) level in Kazakhstan. Reported incident CNRs and prevalence vary by oblast, thus the study investigated which determinants contribute to this regional variation. By analyzing aggregate and individual case records provided by the country’s National Institute of Geography and the National Tuberculosis Program, we compared burdens among oblasts and identified significant risk determinants.

## Methods

### Data Source

Surveillance data from the National Tuberculosis Program (NTP) and the National Institute of Geography (NIG) were obtained for the years 2006 through 2010. NTP surveillance data included all new tuberculosis cases diagnosed and reported to the NTP from January 1, 2006 through December 31, 2010 and tuberculosis and MDR-TB national incidence and prevalence (2006 – 2010) estimates. The NIG provided surveillance data on oblast population estimates (2006 – 2010). De-identified data was obtained in Microsoft Access 2010, Excel 2010, and Word 1997–2003. Standard data security and management procedures were followed to ensure the highest level of data safety. The study was conducted under a protocol approved by the Institutional Review Boards of Columbia University and Nazarbayev University.

As described above, the NTP provides free diagnosis and treatment of TB and MDR-TB. Kazakhstan’s laboratory network holds a Class B rating by the WHO [20]. Routine testing provides data on TB cases, with positive cultures available for a minimum of 35% of notified cases. Drug susceptibility is tested for a minimum of 50% of positive culture cases. The entire notification process delivers a “moderately high degree of representativeness” of the MDR-TB national burden [21]. The NTP defines a TB case as any suspected TB case that has even one positive smear, or a negative smear examination and a clinical and radiographic presentation consistent with TB that responds to drug treatment [20]. MDR-TB is defined as resistance to both isoniazid and rifampicin. The surveillance data utilized for the current analysis does not differentiate between new and retreatment MDR-TB cases.

Individual risk factors recorded by the NTP and included in this analysis are alcohol use, child or youth from a vulnerable group, known diabetes diagnosis, history of illicit drug use, incarceration history within the past two years, migrant status, non-regular uptake of anti-tuberculosis medication (less relevant for new cases), prison system staff member, recent mother (having given birth within one year of diagnosis), registered contact of a TB or MDR-TB case, TB health care staff member, and a category listed as “unknown individual risk factors.” Additionally, categories for employment status included currently incarcerated, detainee, farmer, officer, pensioner, child or youth, prison medical staff, self-employed, student, TB clinic medical staff, worker, unemployed, or other. Individual level data was not available for geocoding beyond the rayon (community) level; therefore, analysis on residential related variables could not be conducted.

### Methods

Descriptive statistical analyses on the oblast and national levels were conducted in Microsoft Excel 2010 and SAS 9.2. National and oblast level incident CNRs and prevalence data presented here were reported directly from the NTP and are assumed to have an underlying Poisson distribution [21,22]. Poisson ratio comparisons for statistically significant associations were conducted on national and oblast level incident CNR and prevalence estimates to test for statistically significant change (p < 0.05) [22,23]. Pearson correlation coefficients were calculated between total cases with known characteristics of incident cases and total new cases registered, by year (p < 0.05) [24,25].

## Results

### Incident CNR and prevalence of TB and MDR-TB

Table 1 presents the incident CNR and prevalence of TB and MDR-TB in Kazakhstan from 2007 to 2010 as reported by the NTP. CNRs of TB and MDR-TB are presented by oblast. In 2007 the CNR of tuberculosis was reported as 126.4 cases per 100,000 and in 2010 that figure significantly declined to 95.3 cases per 100,000 (p = 0.02). Nine out of the fourteen oblasts in Kazakhstan observed a statistically significant decrease in CNR of tuberculosis from 2007 to 2010 (Akmolinskaya,Aktubinskaya, Atyrauskaya, Karagandinskaya, Kostanayskaya, Kyzylordinskaya, Mangystauskaya, North-Kazakhstanskaya, and Pavlodarskaya oblasts). The national prevalence similarly significantly decreased from 283.6 cases per 100,000 in 2007 to 166.3 cases per 100,000 in 2010 (p < 0.01).

**Table 1:** Tuberculosis prevalence and case notification rate in Kazakhstan from 2007 – 2010†

**Variable**	**2007**	**2008**	**2009**	**2010**	**Change 2007 - 2010**
CNR of tuberculosis (per 100,000 population)	126.4	125.5	105.3	95.3	**p = 0.02**
Almatynskaya oblast	53.7	55.7	50.5	49.8	p = 0.35
Akmolinskaya oblast	85.6	92.1	82.5	62.6	**p = 0.03**
Aktubinskaya oblast	138.2	122.1	96.8	87.1	**p < 0.01**
Atyrauskaya oblast	168.1	160.9	124.4	120.8	**p < 0.01**
East-Kazakhstan oblast	129.4	128.5	121.0	122.1	p = 0.32
Karagandinskaya oblast	129.5	114.2	96.6	89.3	**p < 0.01**
Kostanayskaya oblast	141.8	143.8	114.0	108.1	**p = 0.02**
Kyzylordinskaya oblast	167.5	155.8	120.6	110.5	**p < 0.01**
Mangystauskaya oblast	155.7	157.3	123.6	111.8	**p < 0.01**
North-Kazakhstanskaya oblast	154.8	152.9	118.5	96.1	**p < 0.01**
Pavlodarskaya oblast	141.9	138.4	111.2	97.0	**p < 0.01**
South-Kazakhstanskaya oblast	83.7	87.2	77.4	77.3	p = 0.31
West-Kazakhstan oblast	109.6	137.3	106.0	94.3	p = 0.14
Zhambylskaya oblast	100.4	115.3	89.4	86.4	p = 0.15
Prevalence of tuberculosis (per 100,000 population)	283.6	201.4	180	166.3	**p < 0.01**
CNR of multidrug resistant tuberculosis (per 100,000 population)	5.8	8.5	8.5	10.5	p = 0.12
Almatynskaya oblast	4.1	7.7	7.8	13.1	**p = 0.02**
Akmolinskaya oblast	3.2	2.2	7.2	7.0	p = 0.12
Aktubinskaya oblast	3.1	12.0	10.5	8.7	**p = 0.05**
Atyrauskaya oblast	13.2	18.5	16.7	22.8	**p = 0.05**
East-Kazakhstan oblast	8.0	11.2	13.7	15.2	p = 0.07
Karagandinskaya oblast	6.0	6.9	6.3	11.3	p = 0.10
Kostanayskaya oblast	5.2	4.4	12.5	4.0	p = 0.35
Kyzylordinskaya oblast	5.1	13.1	2.7	15.4	**p = 0.01**
Mangystauskaya oblast	15.5	16.8	15.1	11.1	p = 0.20
North-Kazakhstanskaya oblast	5.9	13.2	11.3	12.0	p = 0.07
Pavlodarskaya oblast	6.6	13.3	7.2	9.8	p = 0.21
South-Kazakhstanskaya oblast	5.0	1.7	2.4	4.3	p = 0.41
West-Kazakhstan oblast	7.2	10.0	9.3	13.7	p = 0.08
Zhambylskaya oblast	6.2	15.4	10.4	13.1	p = 0.06
Prevalence of multidrug resistant tuberculosis (per 100,000 population)	54.4	49.8	52.9	61.6	p = 0.25

† NTP Center Statistics.

National MDR-TB incident CNR has conversely increased over the same time period, from 5.8 cases per 100,000 in 2007 to 10.5 cases per 100,000 in 2010 (p = 0.12). Four out of fourteen oblasts observed a statistically significant change in MDR-TB incident CNR (Almatynskaya, Aktubinskaya, Atyrauskaya, and Kyzylordinskaya oblasts). Almatynskaya oblast was one of the four oblasts that did not experience a statistically significant decrease in TB CNR over the same time period. National MDR-TB prevalence increased from 54.4 cases per 100,000 in 2007 to 61.6 cases per 100,000 in 2010 (p = 0.25).

**Figure 1:**
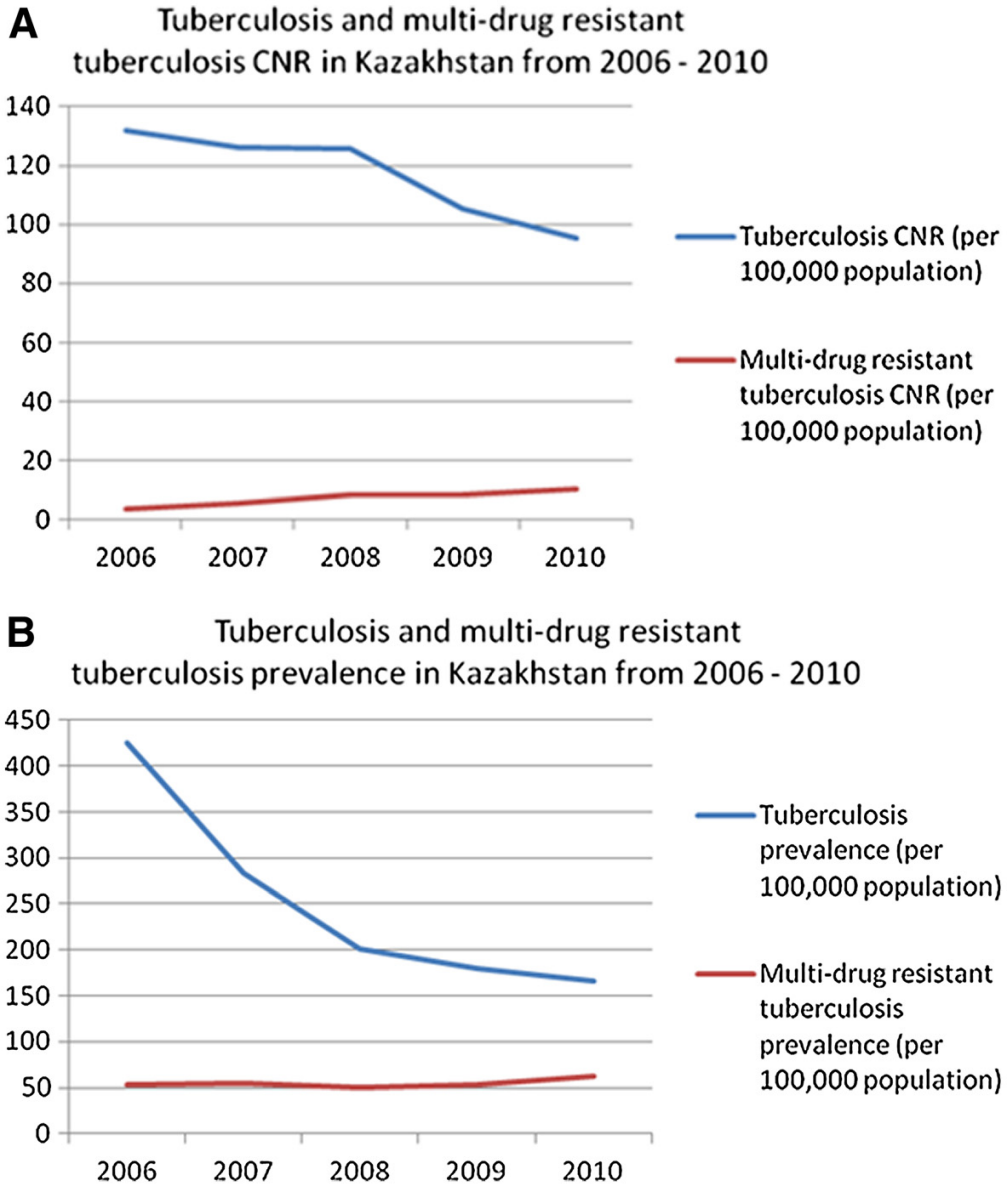
**A: Tuberculosis and multidrug resistant tuberculosis CNRs in Kazakhstan from 2006 – 2010.** Tuberculosis CNR decrease from 2006 to 2010 was statistically significant on a p = 0.05 level. **B**: Tuberculosis and multidrug resistant tuberculosis prevalence in Kazakhstan from 2006 – 2010. Tuberculosis prevalence decrease from 2006 to 2010 was statistically significant on a p = 0.05 level.

Nationally aggregated TB and MDR-TB CNRs and prevalence data was available for 2006. Its inclusion in the analysis supports the observed decrease of TB CNR coupled with the increase in MDR-TB CNR. Figure 1A and 1B compare TB and MDR-TB CNRs and prevalence from 2006 to 2010. While statistically significant decreases in tuberculosis CNR (p < 0.01) and prevalence (p < 0.01) have been achieved, the MDR-TB CNR (p < 0.04) and prevalence (p = 0.22) burdens have increased.

**Figure 2:**
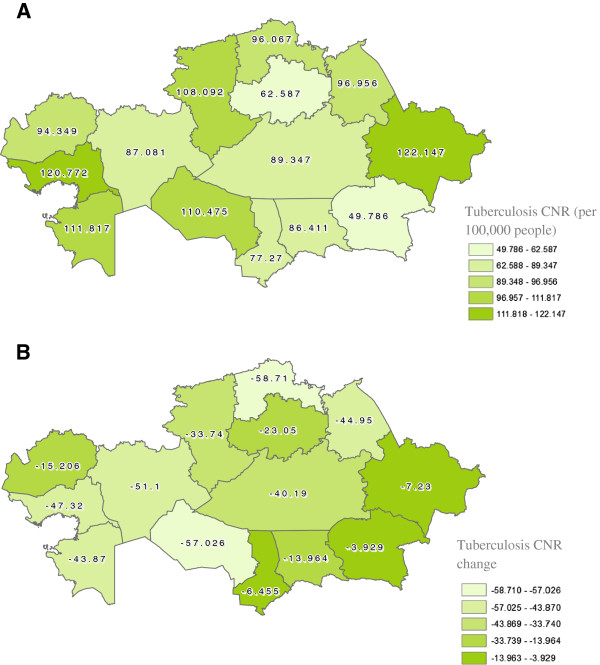
**A: Spatial distribution of tuberculosis case notification rate in Kazakhstan 2010.B**: Spatial distribution of tuberculosis case notification rate change in Kazakhstan from 2007 – 2010. Tuberculosis CNR change was calculated by finding the difference between the 2010 CNR and the 2007 CNR. All oblasts observed a reduction in incidence, and the more negative represent those areas that experienced a larger decrease compared to those that are less negative. Oblasts in the three lightest range and some of the fourth experienced a statistically significant change on the p = 0.05 level.

**Figure 3:**
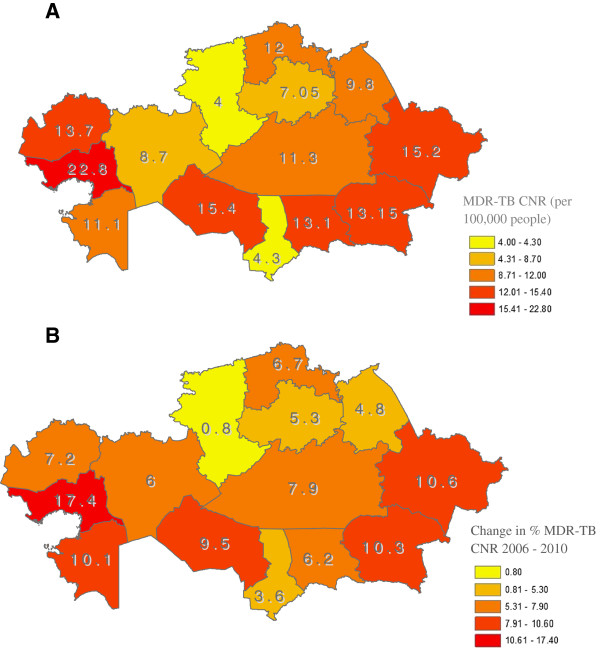
**A: Spatial distribution of MDR-TB case notification rate in Kazakhstan 2010.B**: Spatial distribution of tuberculosis case notification rate change in Kazakhstan from 2006 – 2010**.** Multidrug resistant tuberculosis CNR percentage change was calculated by finding the difference between the 2010 and 2006 CNRs. All oblasts experienced an increase in MDR-TB CNR, and the more positive represent those areas that experienced a larger increase as compared to those that are smaller. More than half the oblasts in the country experienced a statistically significant change on the p = 0.05 level.

Tuberculosis and MDR-TB have different spatial distributions within Kazakhstan (see Figures 2A-Figure 3B). In 2010, Atyrauskaya and East-Kazakhstan oblasts had the highest CNRs of tuberculosis with Almatynskaya and Akmolinskaya oblasts reporting the lowest CNRs. From 2007 – 2010 North-Kazakhstan and Kyzylordinskaya oblasts reported the greatest percentage decrease in CNRs (both p < 0.01). For MDR-TB in 2010, again Atyrauskaya and East-Kazakhstan oblasts were in the highest category of CNRs in the country, with Atyrauskaya oblast reporting the largest percentage increase in MDR-TB CNR (p = 0.05). Kostanayskaya and South-Kazakhstan oblasts reported the lowest CNRs of MDR-TB in the country with Kostanayskaya also having the lowest percentage increase in CNR (p = 0.35).

### Characteristics of new TB cases in Kazakhstan

Table 2 summarizes key characteristics of tuberculosis cases from the NTP registry. Risk factors collected at diagnosis include whether or not the case is a registered contact of another tuberculosis or MDR-TB case (5% and <1% in 2010 respectively), has diabetes (2%, 2010), reports illicit drug or alcohol use (<1% and 2% in 2010 respectively), was incarcerated within the past two years (2%, 2010), is a child or youth from a vulnerable group (1%, 2010), is a staff member in a TB treatment unit or in a prison (<1% for both, 2010), is a recent mother (2%), had non-regular uptake of anti-tuberculosis medication (<1%, 2010), or is a migrant (3%, 2010). New cases in this dataset are first time cases referred to the NTP center at diagnosis. Based on available data, it is impossible to determine if cases labeled as non-regular uptake of anti-tuberculosis medication are done so because they are treatment failures, retreatment cases, or have a historic lack of adherence episode. Most illustrative is that the NTP consistently reports that over 85% of the cases referred indicate that risk factors are “unknown” at diagnosis. The categorization of “unknown risk factor” requires further exploration in subsequent studies. Considering the employment status of notified cases in 2010, most were unemployed (52%), with workers as the next highest category (14%). Other employment categories were represented by less than 10% of respondents.

**Table 2:** Kazakhstan tuberculosis new case characteristics†

**Variable**	**2007 n (%)**	**2008 n (%)**	**2009 n (%)**	**2010 n (%)**
Total new cases registered	18,290	19,311	16,608	15,617
*Risk factors*				
Alcohol use	787 (4%)	639 (3%)	398 (2%)	346 (2%)
Child or youth from vulnerable group	362 (2%)	271 (1%)	237 (1%)	175 (1%)
Diabetes	252 (1%)	284 (1%)	263 (2%)	246 (2%)
Drug use	58	51	47	44
Incarcerated within past 2 years*	70	74	53	52
Migrant	174 (1%)	922 (5%)	444 (3%)	417 (3%)
Non-regular uptake of anti-tuberculosis medication	14	43	23	24
Prison system staff member	31	28	21	18
Recent mother (birth within 1 year)	204 (1%)	397 (2%)	330 (2%)	323 (2%)
Registered contact of a multidrug resistant tuberculosis case	28	62	64	72
Registered contact of a tuberculosis case*	928 (5%)	1034 (5%)	862 (5%)	739 (5%)
Tuberculosis health care staff member	1	1	0	3
Unknown risk factor*	15,883 (87%)	15,953 (83%)	14,173 (85%)	13,451 (86%)
*Socio-economic indicators*				
Employment status				
Currently incarcerated	6	8	5	11
Detainee*	0	0	2	3
Farmer	8	7	8	4
Officer*	1,365 (7%)	1,406 (7%)	1,246 (8%)	1,048 (7%)
Other	1,006 (6%)	1,083 (6%)	748 (5%)	832 (5%)
Pensioner	893 (5%)	994 (5%)	816 (5%)	868 (6%)
Pre-school child	45	160 (1%)	140 (1%)	141 (1%)
Prison medical staff	13	128 (1%)	141 (1%)	106 (1%)
Self-employed	465 (3%)	139 (1%)	92 (1%)	84 (1%)
Student	1,566 (9%) or 975 (5%)	1,522 (8%) or 1,066 (6%)	1,401 (8%) or 967 (6%)	1,293 (8%) or 843 (5%)
Tuberculosis clinic medical staff	13	32	35	30
Unemployed*	9,029 (49%)	10,054 (52%)	8,655 (52%)	8,088 (52%)
Worker*	2,707 (15%)	2,681 (14%)	2,302 (14%)	2,234 (14%)

† NTP registry, percentages are not presented for variables representing less than 1% of cases.

* Variable is statistically correlated with number of new cases of TB at a p = 0.05 level.

Of the 26 characteristics collected, seven were significantly correlated with new TB case notification. These included being a registered contact of a TB case (p = 0.02), being incarcerated within the past two years (p = 0.03), having unknown risk factors (p = 0.03), self-identifying as an officer (p = 0.05), self-identifying as a worker (p = 0.05), self-identifying as unemployed (p = 0.04), and self-identifying as a detainee (p = 0.03).

## Discussion

Kazakhstan has a high rate of tuberculosis overall, and one of the highest rates of MDR-TB in the world. This study provides a comprehensive description of the epidemiology of tuberculosis and MDR-TB in this rapidly developing Central Asian nation. The findings demonstrate that although the overall CNR of tuberculosis has declined significantly over the past five years, the CNR of MDR-TB has risen. It is probable that there is both under- and over-diagnosis of TB cases in Kazakhstan because a substantial proportion of cases are identified clinically and unconfirmed by culture. Some of these cases may represent prior, healed TB with exacerbations of bronchiectasis, for example. In addition, it is likely that the lack of routine access to culture and drug susceptibility testing may result in considerable under-diagnosis of tuberculosis cases overall, and perhaps differential under-diagnosis of drug-resistance. It is quite probable that a low CNR for MDR-TB represents under-diagnosis, and this under-diagnosis is contributing to the spread of MDR-TB in several oblasts. Rapid diagnosis of TB and drug susceptibility testing using the WHO-recommended Cepheid GenExpert is expected in Kazakhstan in late 2012. Cepheid GenExpert should improve detection and treatment of MDR-TB cases.

The national data on CNRs and MDR-TB are not uniformly distributed; Atyrauskaya and Mangystauskaya oblasts present anomalies with large decreases in TB CNRs coupled with comparatively large increases in MDR-TB CNRs. The lack of uniformity in distribution may result from differential socio-environmental factors and a lack of access to treatment. After the FSU collapse, access to anti-tuberculosis chemotherapy became limited and sporadic, and supervision and surveillance of patients with tuberculosis was inadequate. These factors undoubtedly contributed to the proliferation of MDR-TB cases in Russia and FSU republics that is apparent today. Understanding the reasons for the differential socio-environmental factors and lack of access to treatment will be critical to developing effective strategies for controlling TB in Kazakhstan and limiting the proliferation of MDR strains of tuberculosis.

Kazakhstan’s national TB control program classified a high percentage of patients (83% – 87% throughout the study period) as being without identified risk factors for tuberculosis. Such risk factors may be related to undetected TB transmission in the community or in institutional settings. Many instances of nosocomial transmission of tuberculosis, especially of MDR-TB, have been reported around the world, including diverse locations such as New York and Johannesburg, and it is possible that a similar situation has occurred in Kazakhstan. It is also possible that some MDR strains currently present in Kazakhstan are particularly virulent and capable of generating a large number of secondary cases before a new index case is diagnosed. Risks may also be related to selection of the single drug resistant strains induced by previous treatments.

An influx of migrant workers has been attracted to the country because of its rapid development. Migrants typically are at increased risk of tuberculosis as they have limited access to health care services, including anti-tuberculosis chemotherapy, and reside in crowded conditions that favor the rapid transmission of tuberculosis. It is also possible that immigrants are entering Kazakhstan knowing that they have MDR-TB because they perceive that services and treatment are available that are not attainable in their home countries. This could lead to a rise in MDR-TB cases that outpaces the rise in drug-susceptible cases. This explanation is supported by the large increases in MDR-TB prevalence reported in the two oblasts (Atyrauskaya and Mangystauskaya) with the highest immigration rates. Most migrants come to these areas from other high TB incidence Central Asian countries. Previous studies have reported higher risks for MDR-TB in foreign-born individuals [25]. Future studies need to investigate the link between migration and MDR-TB CNRs, taking into account previous treatment.

Another possibility for increasing MDR-TB CNRs is that although DOTS is available for patients reported to the NTP, delayed detection of drug resistance among clinically and microbiologically confirmed cases of tuberculosis limits DOTS effectiveness. Patients may not be receiving ideal therapy in a timely fashion. Use of the Cepheid GenExpert system could ameliorate this situation. GenExpert will be introduced in four regions of Kazakhstan in 2012 with the help of a USAID-funded pilot project. The sustainability of the GenExpert system long-term is yet to be determined. Furthermore, if cases of MDR-TB are not promptly diagnosed, nosocomial transmission of MDR-TB could be playing a substantial role in the rising MDR-TB rates, particularly as the practice in Kazakhstan is to treat all TB patients with prolonged periods of hospitalization.

Positive HIV/AIDS status is an important risk factor related to increasing MDR-TB transmission rates. Kazakhstan, along with other eastern European and Central Asian countries, belongs to the region with the fastest growing HIV epidemic in the world. Several studies noted that HIV-infected patients with TB develop drug-resistance, particularly to rifampicin, in response to erratic administration of chemotherapy [26,27]. This could lead to drug-susceptible cases being converted to drug-resistant cases, even without increasing the total number of TB cases overall [28,29]. Further studies are required, and one is currently underway, to understand the relationship between HIV status and drug-susceptibility in Kazakhstan.

Other aspects of the current study deserve particular comment. Compared with previous research, recent incarceration or employment in a prison was not highly represented as a reported risk factor for new TB cases. This could represent a true improvement in control and treatment programs within the prison system or an artifact of the reporting process.

### Limitations

The study findings represent an analysis of all the data reported to the NTP. However, through a detailed review and analysis of the data provided for this study, various inconsistencies in reporting, variable definitions, and collection procedures were uncovered when comparing NTP data with other published documents. The data might not accurately reflect the diverse collection and reporting methods currently functioning across the country. Divergent MDR-TB CNRs could be reflective of the quality of the NTP or an artifact of diverse testing practices. Atyrauskaya and Mangystauskaya could report higher incidence of MDR-TB because the oblasts may have more financial resources available and impetus to test suspected TB cases than areas such as South-Kazakhstan. These inconsistencies are being investigated through continued collaboration with our study partners at the NTP and NIG. Accurate surveillance and reporting is critical to controlling tuberculosis in any locale.

## Conclusion

In conclusion, this study expands current knowledge of the epidemiology of tuberculosis in Kazakhstan. Notwithstanding the limitations detailed above, understanding the distribution of TB and MDR-TB cases and associated risk factors provides context for future studies. The continuing rise of MDR cases in Kazakhstan and other countries in the FSU should be considered a global health emergency. The findings presented here provide a roadmap to further investigations that can lead to the underlying causes of the problem. Understanding the ‘unknown risk factor’ categorization and reporting practices across the country are two ideal starting points for further research that will yield benefits to the entire public health system in Kazakhstan.

## Abbreviations

WHO: World Health Organization; TB: Tuberculosis; FSU: Former Soviet Union; MoH: Kazakhstan’s Ministry of Health; DOTS: Directly Observed Therapy Short Course; NTP: National Tuberculosis Program; MDR-TB: Multidrug Resistant Tuberculosis; NIG: National Institute of Geography; CNR: Case notification rates.

## Competing interests

The author(s) declare that they have no competing interests.

## Authors’ contributions

AT had full access to all of the data in the study and takes responsibility for the integrity of the data and the accuracy of the data analysis. Study concept and design: AT, NE, SG, NS. Acquisition of data, critical review of intellectual content and final approval of the version to be published: TA, TM, FA, AS, ZZ. Analysis and interpretation of data: SH, SG, NS, NE. Drafting of the manuscript: SH and LB with feedback from SG, NE, NS, AT. Critical revision of the manuscript for important intellectual content: SG, NS, NE, AT. Statistical analysis: SH, SG. Obtained funding: AT, NE, SH. All authors read and approved the final manuscript.

## Pre-publication history

The pre-publication history for this paper can be accessed here:

http://www.biomedcentral.com/1471-2334/12/262/prepub
